# Apoptosis Signal-regulating Kinase 1 promotes Ochratoxin A-induced renal cytotoxicity

**DOI:** 10.1038/srep08078

**Published:** 2015-01-28

**Authors:** Rui Liang, Xiao Li Shen, Boyang Zhang, Yuzhe Li, Wentao Xu, Changhui Zhao, YunBo Luo, Kunlun Huang

**Affiliations:** 1 Laboratory of food safety and molecular biology, College of Food Science and Nutritional Engineering, China Agricultural University, Beijing 100083, P.R. China; 2 School of Public Health, Zunyi Medical University, Zunyi, Guizhou 563003, P.R. China; 3 Department of Nutrition and Food Science, University of Maryland, College Park, MD 20742, USA

## Abstract

Oxidative stress and apoptosis are involved in Ochratoxin A (OTA)-induced renal cytotoxicity. Apoptosis signal-regulating kinase 1 (ASK1) is a Mitogen-Activated Protein Kinase Kinase Kinase (MAPKKK, MAP3K) family member that plays an important role in oxidative stress-induced cell apoptosis. In this study, we performed RNA interference of ASK1 in HEK293 cells and employed an iTRAQ-based quantitative proteomics approach to globally investigate the regulatory mechanism of ASK1 in OTA-induced renal cytotoxicity. Our results showed that ASK1 knockdown alleviated OTA-induced ROS generation and Δψm loss and thus desensitized the cells to OTA-induced apoptosis. We identified 33 and 24 differentially expressed proteins upon OTA treatment in scrambled and ASK1 knockdown cells, respectively. Pathway classification and analysis revealed that ASK1 participated in OTA-induced inhibition of mRNA splicing, nucleotide metabolism, the cell cycle, DNA repair, and the activation of lipid metabolism. We concluded that ASK1 plays an essential role in promoting OTA-induced renal cytotoxicity.

Ochratoxin A (OTA) is a toxic secondary metabolite produced by several species of Aspergillus and Penicillium^1^,^2^. OTA induces a wide range of toxicological effects, including nephrotoxicity, teratogenicity, immunotoxicity, carcinogenicity, and mutagenicity^3^. The kidney represents the main target of OTA, and OTA is suspected to be responsible for human Balkan endemic nephropathy (BEN)^3^,^4^. Due to its ubiquitous presence in a variety of foodstuffs, the complete avoidance of OTA exposure is impossible^5^,^6^. Therefore, understanding the OTA toxification mechanism is of great importance to human and animal health.

Although the mechanism of OTA-induced cytotoxicity has not been fully elucidated, oxidative stress^7^,^8^ and apoptosis^9^,^10^ have been proven to be involved in this process. Furthermore, it has been reported that OTA might regulate cell fate via stimulating Mitogen Activated Protein Kinase (MAPK) family members, including ERK1/2, JNK, and p38 MAPK^4^,^11^,^12^,^13^. MAPKs are evolutionarily conserved serine/threonine kinases that respond to various chemical and physical stresses and play essential roles in cell survival and adaptation^14^. The activity of MAPK is regulated through a three-tiered cascade: MAP kinase kinase kinases (MAPKKKs, MAP3Ks) phosphorylate and activate MAP kinase kinases (MAPKKs, MAP2Ks), and MAP2Ks subsequently phosphorylate and activate MAPKs^15^. Apoptosis signal-regulating kinase 1 (ASK1) is an MAP3K family member that activates both the MKK4/MKK7-JNK and MKK3/MKK6-p38 pathways^16^. ASK1 plays a pivotal role in oxidative stress- and endoplasmic reticulum stress-induced cell death^17^,^18^,^19^. However, the role of ASK1 in OTA-induced cytotoxicity is poorly understood.

Since RNA interference (RNAi) was discovered in *Caenorhabditis elegans* in 1998^20^, RNAi has become a powerful method for the analysis of signal transduction pathways. It has been applied to a wide variety of experimental scales, ranging from the discovery and validation of targets to the analysis of protein actions^21^. However, the global-scale quantification of specific proteins is restricted due to the limited availability of antibody-based protein quantification methods. Bonaldi et al.^22^ conducted a SILAC-based high throughput quantitative proteomic analysis following the silencing of a specific gene, paving the way for investigation of the global impact of RNAi on protein outcomes. Abdrakhmanova et al.^23^ made a step forward by successfully combining RNAi with iTRAQ-based quantitative proteomics, which is a more accurate quantification method with relatively high sensitivity and reproducibility^8^,^24^.

In the present study, we combined RNAi of ASK1 with an iTRAQ-based quantitative proteomics approach to globally profile the function of ASK1 in OTA-induced renal cytotoxicity. We performed a stable knockdown of ASK1 in the human embryonic kidney (HEK293) cell line and compared the proteome between ASK1 knockdown cells and scrambled cells following OTA treatment. In summary, this study, for the first time, showed the function of ASK1 in OTA-induced renal cytotoxicity using a combination of RNAi technology and iTRAQ-based quantitative proteomics.

## Results

### OTA induced ASK1 activation

**Figure 1:**
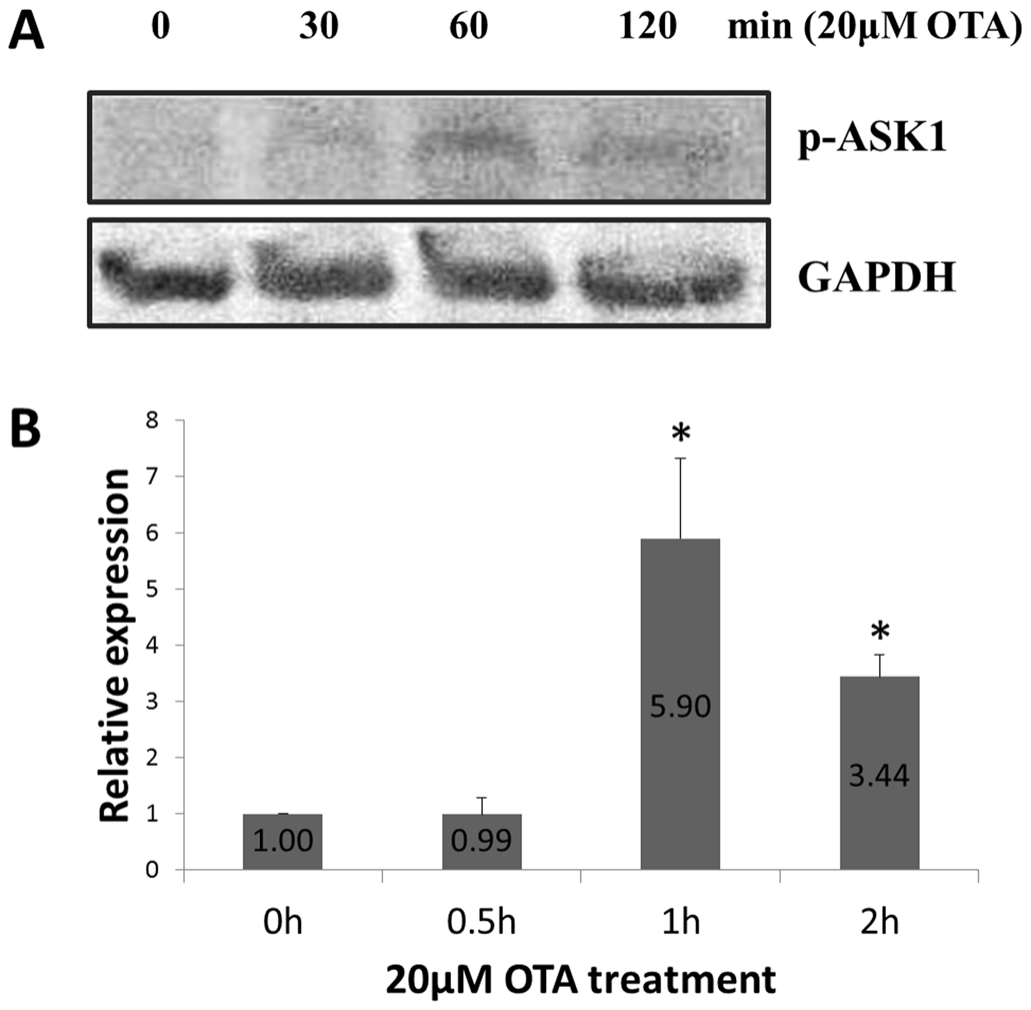
ASK1 was activated by OTA. (A): HEK293 cells were treated with 20 μM OTA for the indicated times, and western blot was used to detect the phosphorylation (Thr838) of ASK1. (B): Relative expression of p-ASK1 after 20 μM OTA treatment was shown as total gray value using BandScan software. The p-ASK1 expression in 0 h was normalized to 1. Asterisk (*) indicates significant differences (*p* < 0.05). Gels were run under the same experimental conditions while images of western blots displayed in cropped format.

Since ASK1 was discovered by Ichijo et al. in 1997^25^, it has drawn much attention for its role in cell apoptosis. ASK1 plays a key role in oxidative stress-induced apoptosis through Thr838 phosphorylation^26^,^27^. Because OTA is capable of inducing oxidative stress and apoptosis, we speculated that ASK1 might be involved in OTA-induced apoptosis. Western Blot analysis of ASK1 phosphorylation confirmed this hypothesis. As shown in Figure 1, ASK1 activity reached its peak at 1 h following OTA treatment and then decreased with the duration of OTA treatment.

### Confirmation of RNA interference efficiency

**Figure 2:**
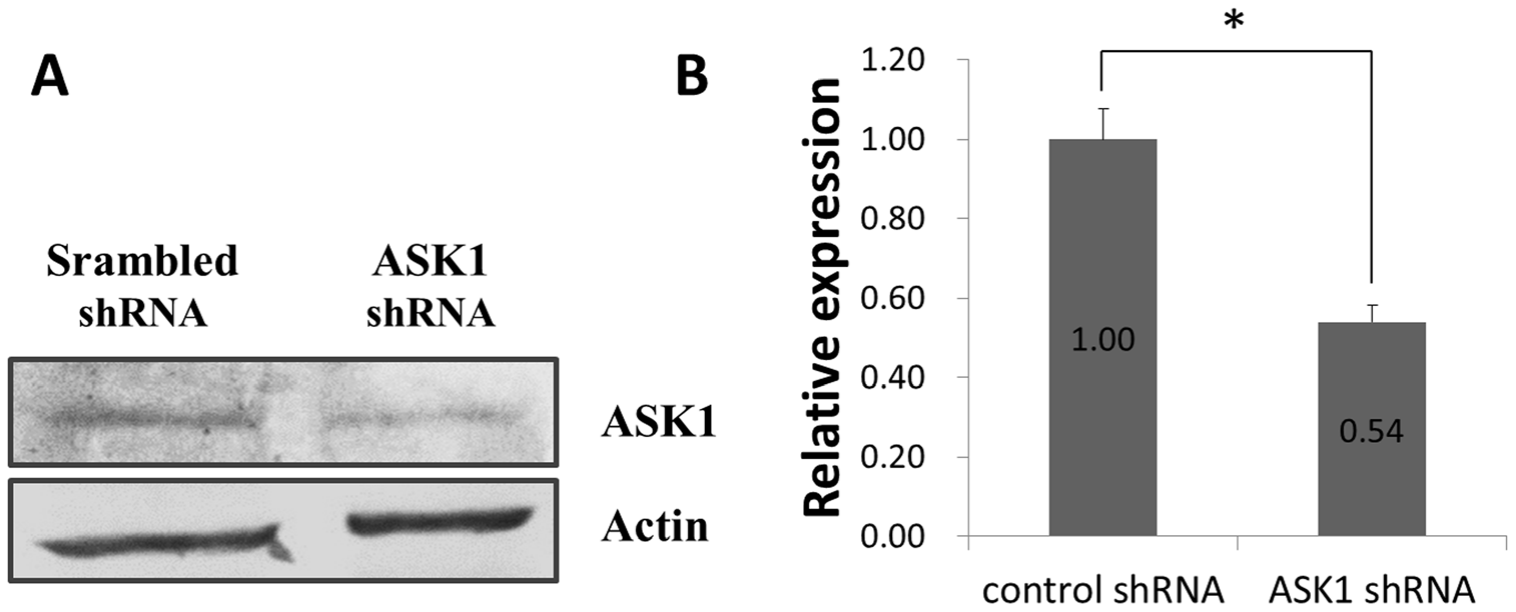
Confirmation of ASK1 knockdown efficiency. (A): Western Blot validation of ASK1 expression in knockdown cells versus scrambled cells. (B): Relative expression of ASK1 in knockdown cells versus scrambled cells was shown as total gray value using BandScan software. The ASK1 expression of scrambled cells was normalized to 1. Asterisk (*) indicate significant differences (*p* < 0.05). Gels were run under the same experimental conditions while images of western blots displayed in cropped format.

To further investigate the role of ASK1 in OTA-induced renal cytotoxicity, we knocked down ASK1 expression using RNA interference. The interference efficiency of ASK1 knockdown cells versus scrambled cells was confirmed by Western blot. As shown in Figure 2, ASK1 shRNA transfection markedly reduced the expression of ASK1 to approximately 54% compared with that of cells transfected with scrambled shRNA.

### ASK1 knockdown desensitized cells to OTA

**Figure 3:**
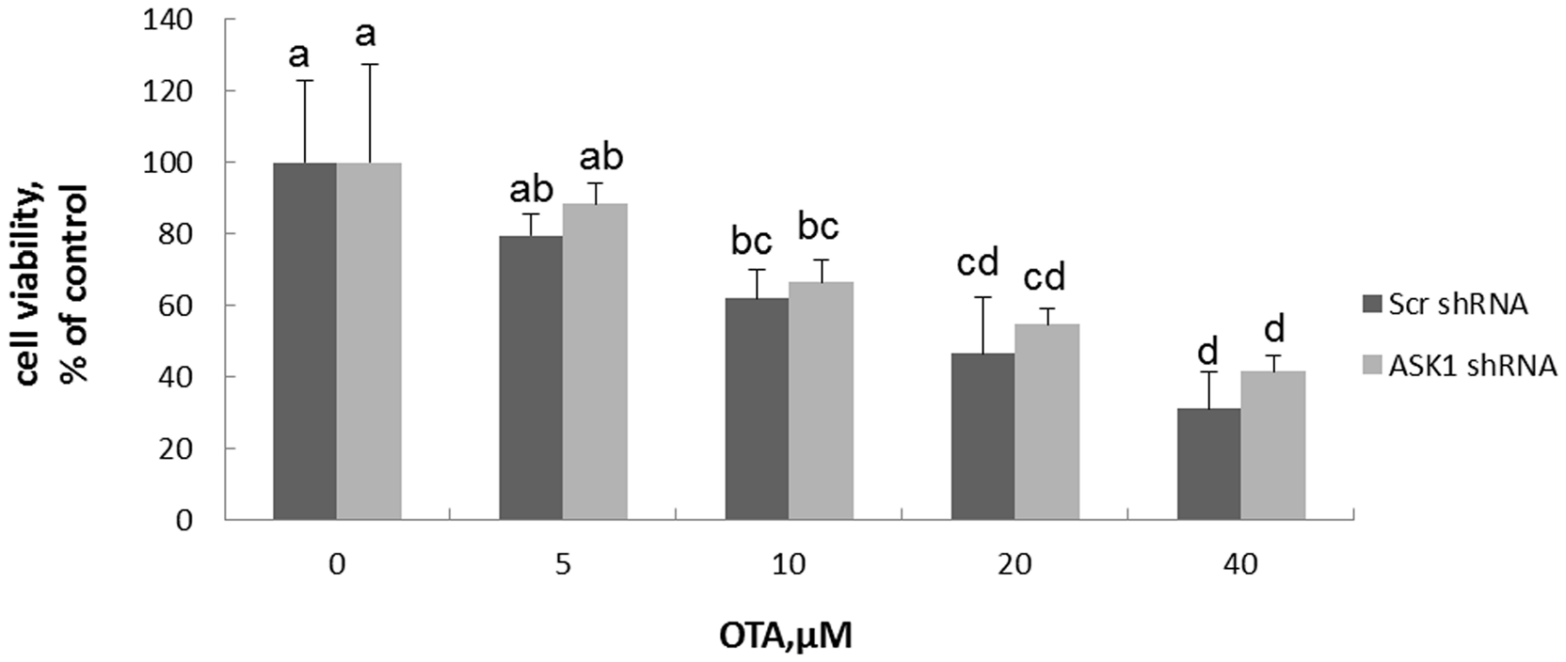
Cell viability of HEK293 cells in response to OTA treatment. Scrambled cells and ASK1 knockdown cells were exposed to increasing concentration of OTA for 24 h and determined by WST-8 staining. Different lowercase letters indicate significant differences (*p* < 0.05).

The cell viability of ASK1 knockdown and scrambled cells after 24 h exposure to increasing concentrations of OTA were determined by WST-8 staining. As shown in Figure 3, OTA treatment caused a decrease of cell viability in a dose-dependent manner; 20 μM OTA treatment caused the cell viability to decrease to 46.4% and 54.7% in scrambled and ASK1 knockdown cells, respectively (Figure 3). Because a dose of 20 μM was close to the IC_50_ for both cell lines, it was chosen for the following study. Interestingly, the cell viability of ASK1 knockdown cells after OTA treatment was slightly higher than that of scrambled cells. Sturchler et al. showed that ASK1 overexpression promoted cell death in HEK293 cells^28^. Our results showed, from another point of view, that ASK1 has a promoting role in OTA-induced cell death.

### ASK1 knockdown reduced OTA-induced ROS generation

**Figure 4:**
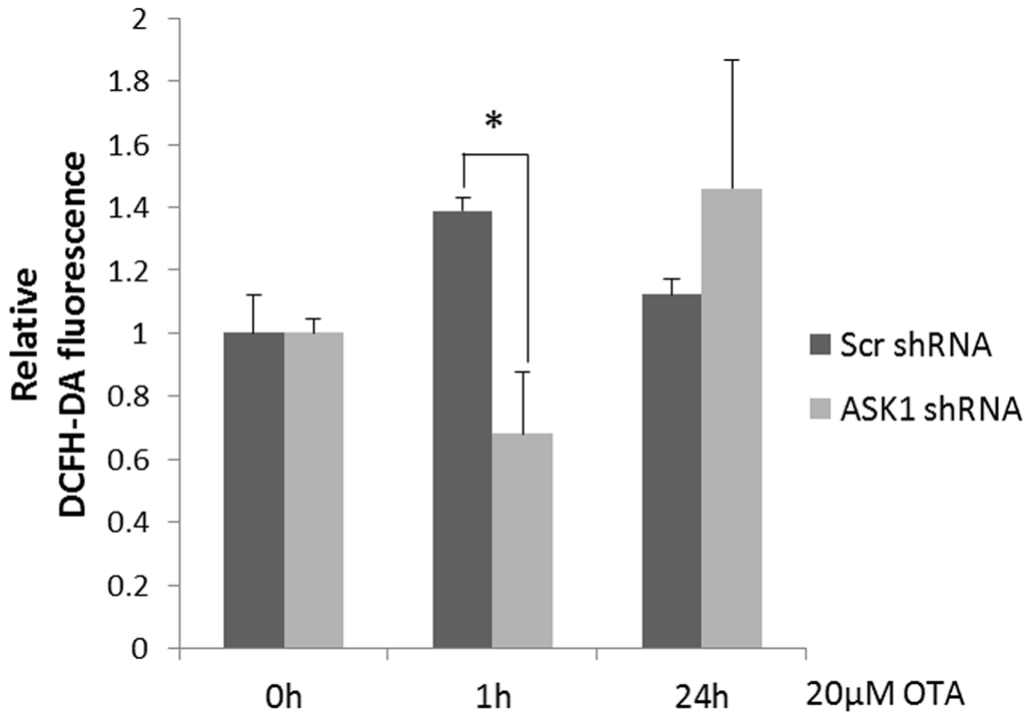
ROS generation of HEK293 cells in response to OTA treatment. Scrambled cells and ASK1 knockdown cells were exposed to 20 μM of OTA for 1 h or 24 h. Cells were stained by DCFH-DA fluorescent probe and determined by flow cytometry. Asterisk (*) indicate significant differences (*p* < 0.05).

It is well documented that ASK1 can be activated by oxidative stress^29^, but how ASK1 affects ROS generation is unknown. Therefore, we examined ROS generation in scrambled and ASK1 knockdown cells in response to 1 h or 24 h of OTA treatment using DCFH-DA fluorescent staining. As shown in Figure 4, OTA treatment markedly increased ROS generation in scrambled cells. However, ASK1 knockdown alleviated this effect at 1 h after OTA treatment. No significant difference was observed between scrambled cells and knockdown cells at 24 h. It has been reported that the targeting of JNK to the mitochondria enhances ROS production through oxidant generation by mitochondrial complex^30^,^31^. Our results suggested that ASK1 enhances ROS production at the transient time point, possibly due to the downstream activation of JNK.

### ASK1 knockdown alleviated OTA-induced Δψm loss

**Figure 5:**
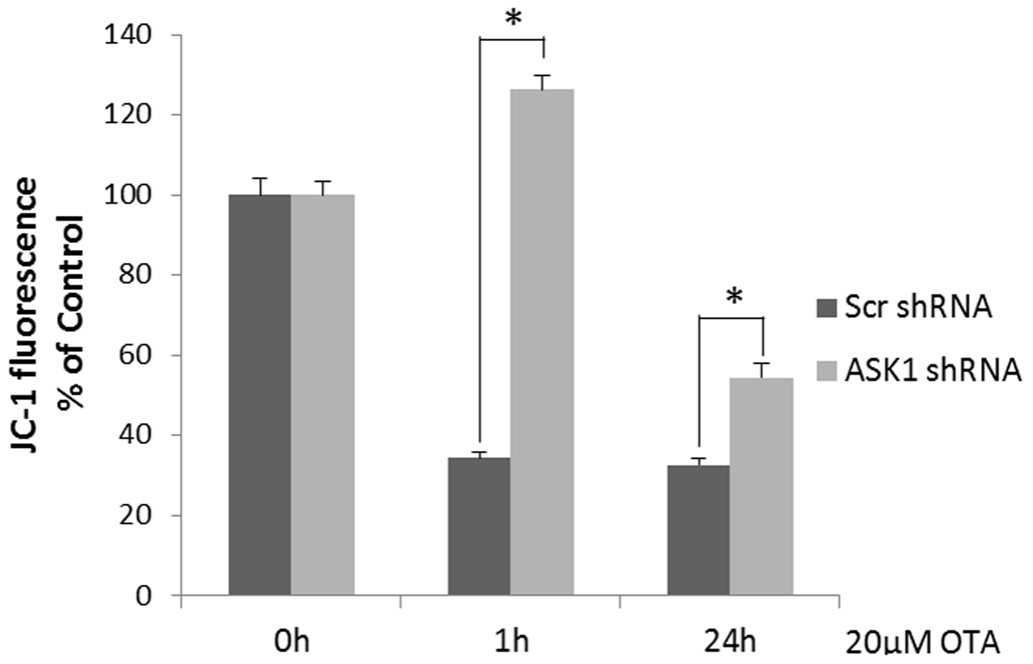
Mitochondrial membrane potential (Δψm) of HEK293 cells in response to OTA treatment. Scrambled cells and ASK1 knockdown cells were exposed to 20 μM of OTA for 1 h or 24 h. Cells were stained by JC-1 and fluorescence was determined by microplate reader. Δψm was expressed as the ratio of red fluorescence over green fluorescence. Asterisk (*) indicate significant differences (*p* < 0.05).

Mitochondrial membrane potential (MMP, Δψm) loss is a symbolic feature of early apoptosis^32^. We measured the Δψm of scrambled and ASK1 knockdown cells in response to 1 h or 24 h of OTA treatment. As shown in Figure 5, OTA exposure significantly reduced the Δψm in HEK293 cells. No significant difference was observed between transient (1 h) and sustained (24 h) OTA-induced Δψm loss. The Δψm loss was significantly less in ASK1 knockdown cells compared with that in scrambled cells. These results suggested that ASK1 might promote OTA-induced mitochondrial membrane potential loss.

### iTRAQ profiling of differentially expressed proteomes

**Figure 6:**
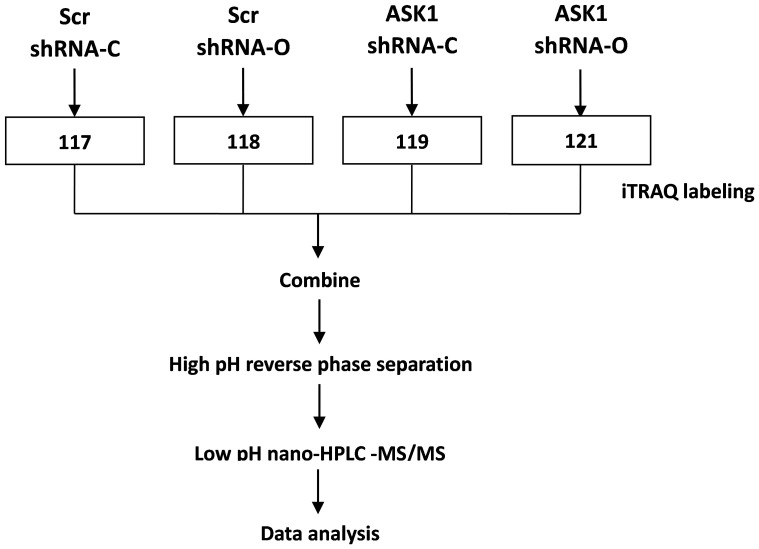
Schematic representation of the experimental design for iTRAQ labeling.

**Figure 7:**
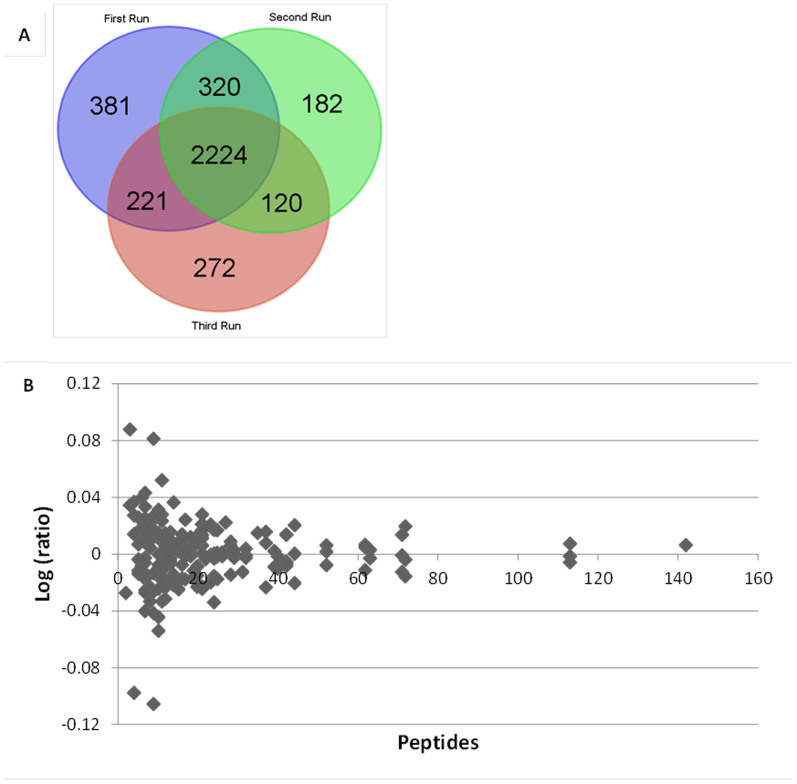
An overview of differentially expressed proteome. (A): Venn diagram depicted the overlap of proteins identified by iTRAQ measurements among three biological replicates. (B): Whetton's plot. Log ratios for all proteins in the OTA treatment versus control were plotted against the number of peptides.

To investigate the global effect of ASK1 on OTA-induced renal cytotoxicity, we performed iTRAQ-based quantitative proteomics on scrambled cells and ASK1 knockdown cells, with or without 20 μM OTA treatment for 24 h (Figure 6). There were 3146, 2846 and 2837 nonredundant proteins (unused protein score ≥ 1.3) identified for three biological replicates, respectively, among which 2224 proteins overlapped (Figure 7A). To assess the variance in the iTRAQ quantification experiments, we introduced a statistical approach described by Ferret-Bernard et al.^33^ Log ratios obtained by comparing differentially expressed proteins (*p* < 0.05) in response to OTA versus the control were plotted against the number of identified peptides (Figure 7B). The extent to which the values deviate from unity is an indication of the variance. We chose a |log (ratio)| value of 0.08 as the threshold, which corresponds to ratios of 1.2 and 0.83, because the vast majority of proteins were within this range (nearly 99%). With this filter, we identified 33 and 24 significantly altered proteins in scrambled cells (118:117, OTA treatment versus control) and ASK1 knockdown cells (121:119, OTA treatment versus control), respectively (Tables 1 and 2).

### Effect of ASK1 on OTA-induced proteomic changes

**Figure 8:**
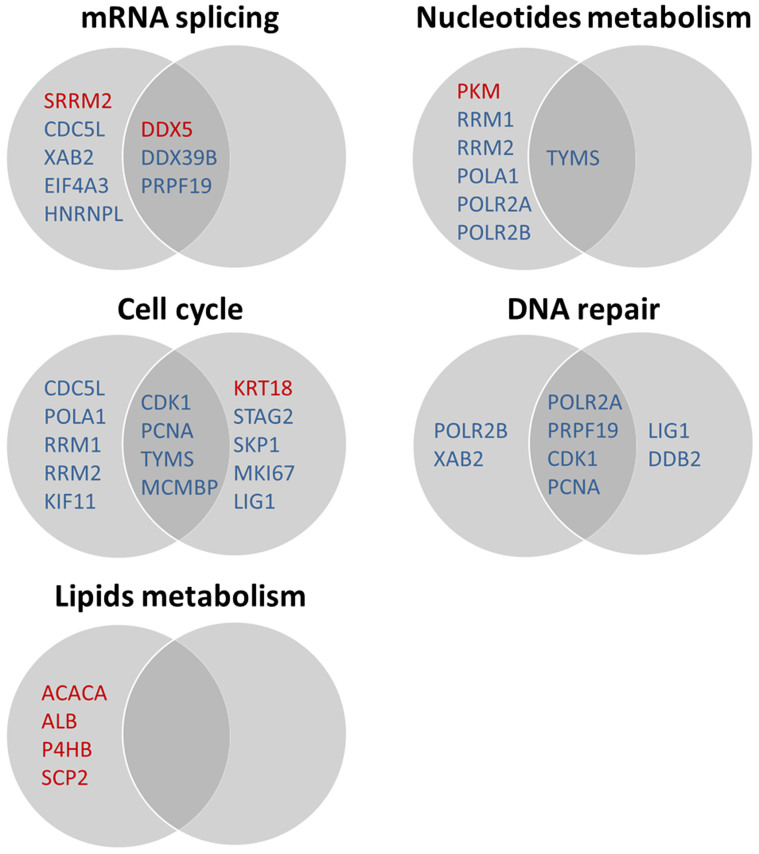
Proteins classified into distinct pathway categories using DAVID functional annotation and UniProt annotation. For each Venn diagram, Left circle indicates differentially expressed proteins in scrambled cell group (OTA treatment versus control) and right circle indicates those in ASK1 knock-down cell group (OTA treatment versus control). The overlapping section indicates proteins both appeared in the two cell groups. Protein names in red represent up-regulated proteins and those in blue represent down-regulated proteins.

As shown in Tables 1 and 2, the expression of 33 proteins was significantly altered in scrambled cells in response to OTA treatment, among which 13 were up-regulated and 20 were down-regulated. Twenty-four proteins were differentially expressed in ASK1 knockdown cells, among which 8 were up-regulated and 16 were down-regulated. All of the proteins were classified according to annotations from the UniProt knowledge base and the GO database. Pathways associated with these proteins were elucidated according to REACTOME_PATHWAY using DAVID functional annotation^34^ (Figure 8). In general, sustained OTA exposure (24 h) exerted influences on mRNA splicing, nucleotide metabolism, the cell cycle, DNA repair, and lipid and lipoprotein metabolism, and ASK1 was implicated in all of these metabolic pathways.

#### mRNA splicing

As shown in Figure 8, OTA treatment down-regulated most of the proteins related to the spliceosome pathway, including EIF4A3, DDX39B, CDC5L, XAB2 and PRPF19. CDC5L and XAB2 are also involved in transcription. HNRNPL is a component of the heterogeneous nuclear ribonucleoprotein (hnRNP) complexes, which provide substrates for pre-mRNA processing events, and is a critical inducible regulator of CD45 alternative splicing^35^. As for the up-regulated proteins, DDX5 and SRRM2 also participate in transcription or the regulation of pre-mRNA splicing. Interestingly, although DDX5 has been reported to be a transcriptional activator, it also functions as a repressor in a promoter-specific manner^36^. Additionally, SRRM2 (also known as SRm300) must form a complex with SRm160 to function as a splicing coactivator. However, the specific depletion of SRm300 does not prevent the splicing of pre-mRNAs, indicating that SRm160 might be the more critical component of the SRm160/300 coactivator^37^. Our results indicated that OTA affects mRNA splicing and transcription, possibly via regulating associated proteins.

Among the 8 differentially expressed proteins, only DDX5, DDX39B and PRPF19 were not affected by ASK1 knockdown. This suggested that ASK1 knockdown partially protected HEK293 cells from OTA-induced mRNA splicing impairment.

#### Nucleotide metabolism

Six proteins involved in nucleotide metabolism were down-regulated by OTA (Figure 8). Among them, RRM1 and RRM2 catalyze the biosynthesis of deoxyribonucleotides from the corresponding ribonucleotides^38^. POLA1, POLR2A and POLR2B play essential roles in pyrimidine or purine metabolism, and TYMS contributes to the de novo mitochondrial thymidylate biosynthesis pathway^39^. Interestingly, PKM, which catalyzes the inter-conversion of ATP and ADP in purine metabolism, was up-regulated upon OTA treatment. However, PKM plays a much more critical role in glycolysis, and overexpression of PKM also contributes to tumorigenesis^40^. Our results generally suggested that OTA inhibits nucleotide metabolism, which might further affect DNA biogenesis and cell proliferation.

Similarly to the role of ASK1 in mRNA splicing, ASK1 knockdown prevented the alteration of 6 proteins (PKM, RRM1, RRM2, POLA1, POLR2A and POLR2B), and partially alleviated the down-regulation fold change of TYMS (from 0.290 to 0.376, *p* < 0.05). These results indicated the participation of ASK1 in OTA-induced nucleotide metabolism impairment.

#### Cell cycle

All 9 proteins involved in the cell cycle were down-regulated by OTA (Figure 8). Among these proteins, CDC5L has been shown to act as a positive regulator of cell cycle G2/M progression^41^; CDK1 plays a key role in promoting the G2-M transition, as well as G1 progression and the G1-S transition^42^. KIF11 is a motor protein required for establishing a bipolar spindle during mitosis^43^. POLA1, PCNA, RRM1, RRM2, TYMS and MCMBP are key players in DNA replication during S phase in the cell cycle. The down-regulation of these proteins suggested that OTA suppresses the cell cycle, especially DNA replication.

ASK1 knockdown prevented the OTA-induced down-regulation of 5 proteins (CDC5L, POLA1, RRM1, RRM2 and KIF11) and alleviated the down-regulation of 4 proteins, of which 3 were significantly different (CDK1 from 0.646 to 0.732, PCNA from 0.652 to 0.681, and TYMS from 0.290 to 0.376, *p* < 0.05). In addition, ASK1 knock-down resulted in the up-regulation of KRT18 and down-regulation of STAG2, SKP1, MKI67 and LIG1. Among them, KRT18 is associated with 14-3-3 protein in vitro in a phosphorylation- and cell cycle-dependent manner; this association occurs during the S/G2/M phases of the cell cycle, when KRT18 become hyperphosphorylated^44^. SKP1 (also known as p19) is a cyclin-dependent kinase inhibitor that inhibits CDK4/6 and controls cell cycle exit^45^. The alteration of these two proteins indicates that ASK1 has an inhibitory role in the cell cycle. However, the down-regulation of STAG2, MKI67 and LIG1, all of which are required for DNA replication or maintaining cell proliferation, indicates that ASK1 also has a promoting role in the cell cycle to some extent.

#### DNA repair

Six proteins related to DNA repair were down-regulated by OTA (Figure 8). Among them, POLR2A, POLR2B, and PCNA play critical roles in nucleotide excision repair (NER)^46^. PCNA also plays a key role in the oxidative DNA damage response and the promotion of post-replication repair^47^. XAB2 is involved in transcription-coupled repair (TCR)^48^. PRPF19 plays a role in DNA double-strand break (DSB) repair and is a component of the PSO4 complex, which is required in the DNA interstrand cross-link (ICL) repair process^49^. CDK1 was inactivated upon DNA damage to stop the cell cycle and genome replication at the G2 checkpoint, thus facilitating DNA repair^50^. It has been shown that OTA induces oxidative DNA damage in various cell lines^8^,^51^. Our results further demonstrated that OTA-induced DNA damage might partly arise from the impaired DNA repair system through the down-regulation of key proteins involved in this system.

Among the 6 altered proteins, 2 (POLR2B and XAB2) were reversed by ASK1 knockdown, and 2 (CDK1 and PCNA) were alleviated in their fold changes. ASK1 knockdown also resulted in the down-regulation of LIG1 and DDB2, both of which are also involved in NER. DDB2 variants from HeLa cells, mostly D1 and D2, were shown to be dominant negative inhibitors of DNA repair^52^. These results suggested that ASK1 has an inhibitory role in DNA repair.

#### Lipid and lipoprotein metabolism

Four proteins in lipid and lipoprotein metabolism were up-regulated upon OTA treatment (Figure 8). Among them, ACACA catalyzes the rate-limiting reaction in the biogenesis of long-chain fatty acids^53^, P4HB acts as a structural subunit of microsomal triacylglycerol transfer protein (MTTP) and SCP2 mediates the transfer of phospholipids between membranes^54^,^55^. ALB is the main protein of plasma and has a high binding capacity for fatty acids, hormones and drugs. No research has been conducted to show whether OTA has an effect on lipid biogenesis and transfer, whereas studies have shown that OTA could induce lipid peroxidation^56^. The up-regulation of lipid metabolism-relevant proteins might be a response mechanism to protect cells against lipid peroxidation. Preventing the up-regulation of these proteins by ASK1 knockdown indicated the involvement of ASK1 in the OTA-induced enhancement of lipid metabolism.

#### Proteins involved in oxidative stress and apoptosis

Because ASK1 is stimulated by oxidative stress and contributes largely to the regulation of apoptosis^15^, we further studied the proteomics data for proteins involved in oxidative stress and apoptosis according to the UniProt database. Three proteins (P4HB, DHRS2 and Prx4) were involved in oxidative stress, and 3 (CDK1, PKM and DHRS2) were involved in the regulation of apoptosis (Tables 1 and 2).

Among the proteins involved in oxidative stress, P4HB (protein disulfide isomerase A1) is a multifunctional protein that catalyzes the formation and rearrangement of disulfide bonds. It was also shown to function in the maintenance of cell redox homeostasis and oxidative protein folding^57^. Intriguingly, P4HB is a thioredoxin (Trx) superfamily protein containing two active Trx domains and is a primary inhibitor of ASK1 in its reduced form^15^,^58^. The oxidizing redox condition in the cell favors the formation of disulfide bonds, leading to the oxidation of reduced Trx into oxidative Trx, whereas P4HB is reduced to an inactive form. This might explain why P4HB is up-regulated upon OTA treatment and how ASK1 is activated in response to OTA treatment.

DHRS2 belongs to the SDR family and was found to possess a protective role against apoptosis induced by oxidative stress^59^. Thus, we assumed that OTA-induced DHRS2 down-regulation could partially result in cell apoptosis; however, ASK1 had little effect on DHRS2 expression.

ASK1 knockdown increased the expression of Prx4, indicating that ASK1 has an inhibitory role on Prx4. Prx4 is an antioxidant enzyme that belongs to the peroxiredoxin family. Prx4 was reported to play a regulatory role in the activation of the transcription factor NF-kappaB^60^. In addition, it was reported that the inhibition of NFκB allows not only prolonged JNK activation but also amplification of ROS stress due to decreased antioxidant transcription^61^,^62^. Taken together, ASK1 might amplify ROS generation through the inhibition of Prx4 and, subsequently, NFκB; this is in accordance with the ROS assay results mentioned above (Figure 4).

As for the proteins related to cell death, CDK1 and DHRS2, both of which are involved in the negative regulation of apoptosis, were down-regulated by OTA, and PKM, which plays a general role in caspase-independent cell death, was up-regulated by OTA. These results suggested that OTA might induce cell apoptosis through the regulation of these proteins. Additionally, ASK1 knockdown reversed the up-regulation of PKM and alleviated the down-regulation fold change of CDK1, confirming the role of ASK1 in OTA-induced cell death.

### Verification of iTRAQ data by Western blot

**Figure 9:**
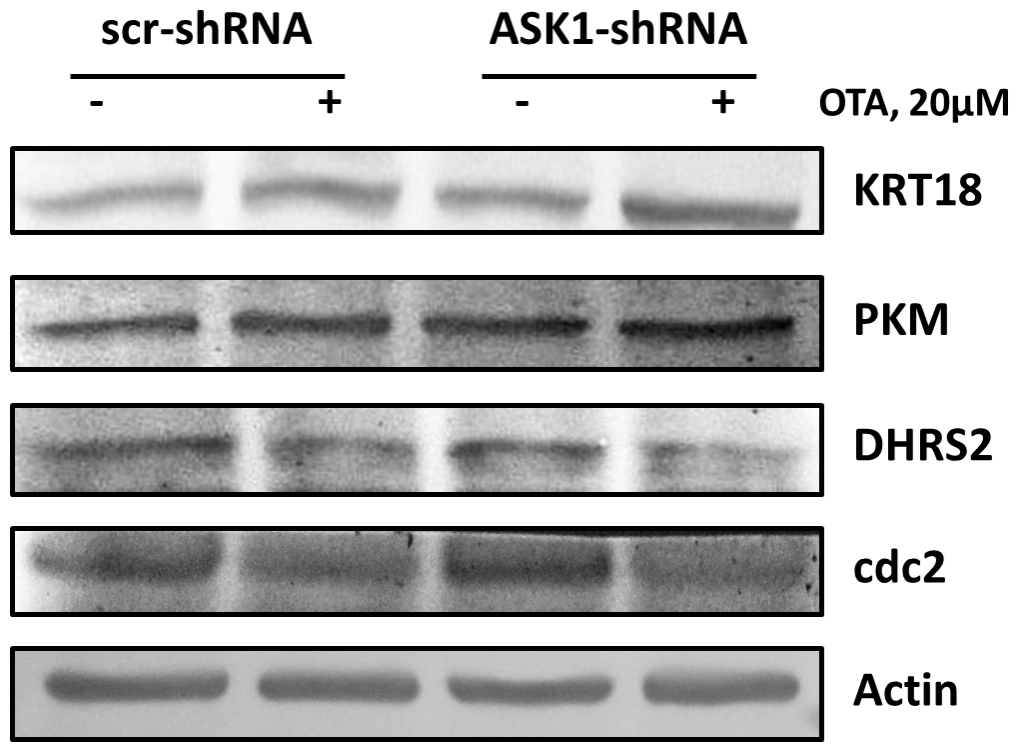
Validation of iTRAQ data by Western Blot. Four proteins were selected to validate the alteration trend using Western Blot. Gels were run under the same experimental conditions while images of western blots displayed in cropped format. KRT18 was up-regulated in ASK1 knockdown cell group and PKM was up-regulated in scrambled cell group compared with control (no drug treatment). CDK1 and DHRS2 were both down-regulated in scrambled cell group as well as ASK1 knockdown cell group compared to control. Actin was used as a loading control.

Four differentially expressed proteins identified in the proteomics data were validated by Western blotting (Figure 9). CDK1 (cdc2) and KRT18 were involved in the cell cycle process, and PKM1/2 and DHRS2 were involved in the regulation of cell death. KRT18 was up-regulated in ASK1 knockdown cells, and PKM was up-regulated in scrambled cells, which was in accordance with the iTRAQ results. In addition, CDK1 and DHRS2 were both down-regulated independently of ASK1, which was also in agreement with the proteomics data. These results confirmed the reliability and accuracy of the iTRAQ-based proteomics technique.

## Discussion

ASK1 has been shown to be an important regulator of oxidative stress-induced cell death and has drawn much attention in apoptosis research^17^. OTA induces a variety of cytotoxic effects, including oxidative stress and apoptosis^7^,^9^. However, to the best of our knowledge, no research has been performed to study the role of ASK1 in OTA-induced cytotoxicity. This study proposed that ASK1 contributes to OTA-induced cell death and impacts other cell signaling or metabolic pathways, as observed in the iTRAQ-based proteomics results.

As oxidative stress is induced by OTA and is one of the major triggers of ASK1 activation, we asked whether ASK1 has a reverse effect on oxidative stress. Intriguingly, although ASK1 is clearly activated by oxidative stress^17^, our result showed that ASK1 is also capable of amplifying ROS generation (Figure 4), probably due to the generation of oxidants and the decreased expression of transcription factors of certain antioxidants. JNK silencing mediated the amplification of ROS production during stress, but p38 silencing had no effect on ROS production^31^. Thus, we speculate that the role of ASK1 in ROS production is mainly through JNK activation.

The loss of Δψm is an apoptotic signal that precedes DNA fragmentation in various cell types and is thus considered a sign of early apoptosis^63^. Our results suggested that ASK1 is required in the breakdown of Δψm and initiation of the mitochondrial-dependent intrinsic apoptotic pathway (Figure 5). This was in accordance with a previous report showing that the early generation of ROS and subsequent signaling through the ASK1 and JNK pathway initiated irreversible intracellular changes, including caspase-3 activation and mitochondrial membrane potential loss, thus contributing to the induction of the death process^64^.

Existing researches of the influence of OTA on metabolic pathways have focused on oxidative stress, apoptosis, cell cycle arrest, DNA damage and repair, and lipid peroxidation^8^,^56^,^65^. Our proteomics data revealed new metabolic pathways affected by OTA such as mRNA splicing, nucleotide metabolism, and lipid metabolism (especially lipid biosynthesis and transfer). The prominent cause of OTA-induced DNA damage was the formation of DNA adducts initiated by the covalent binding of carcinogens to DNA^3^. However, our proteomics data suggested that OTA-induced DNA damage might also be caused by the inhibition of nucleotide biosynthesis. For instance, RRM1 and RRM2 catalyze the biosynthesis of deoxyribonucleotides, and TYMS contributes to the de novo mitochondrial thymidylate biosynthesis. Blocking the formation of the four basic DNA substrates might result in unscheduled DNA synthesis and replication. Angelika et al.^66^ reported that low concentrations of OTA (750 nM–1 μM) caused the dose-dependent induction of DNA repair in rat hepatocyte and porcine urinary bladder epithelial cell cultures. However, based on our proteomics data, a higher concentration of OTA (20 μM) resulted in the inhibition rather than the induction of DNA repair, which is evidenced by the down-regulation of POLR2A, POLR2B, and PCNA in nucleotide excision repair (NER), XAB2 in transcription-coupled repair (TCR), and PRPF19 in double-strand break (DSB) repair. Stefan et al.^67^ reported that OTA-induced DNA damage is increased by blocking repair mechanisms in MDCK cells. Taken together, OTA not only induces DNA damage per se but also impairs DNA repair mechanisms, which further aggravates the aftermath of DNA damage.

The down-regulation of proteins involved in mRNA splicing implied that OTA also induces damage at the RNA level, in addition to DNA damage. Pre-mRNA splicing is one of the three major pre-mRNA processing events and can occur post-transcriptionally or co-transcriptionally^68^. Additionally, the down-regulated protein HNRNPL is a critical inducible regulator of alternative splicing, which generates different mRNAs encoding distinct protein products, including those involved in apoptotic cell death^69^. Altogether, our results suggested that OTA blocks the formation of mature mRNA and subsequently affects the translation and expression of critical proteins.

Hibi et al.^65^ fed male rats with 5 ppm OTA for 4 weeks and observed an up-regulation of cell cycle-related genes. However, our proteomics data showed that the cell cycle was inhibited by OTA. For instance, among all of the down-regulated proteins, CDC5L acts as a positive regulator of G2/M progression, CDK1 plays essential roles in promoting the G2-M transition, G1 progression and the G1-S transition, and KIF11 is required to establish a bipolar spindle during M phase. The inconsistency between the results might arise from dose differences and variations between in vivo and in vitro experiments. OTA might be partially metabolized in vivo, thereby decreasing its toxicity. Last but not least, OTA increased the expression of proteins involved in lipid biosynthesis (ACACA) and lipid transfer (P4HB and SCP2). OTA is capable of inducing lipid peroxidation^56^, and the elevation in lipid metabolism might be a response mechanism for cells to adapt to the severe environment.

ASK1 silencing changed almost all of the proteins in the OTA-induced inhibition or activation of the above pathways (Figure 8). Among them, the alteration of proteins involved in nucleotide and lipid metabolism was completely offset by ASK1 silencing. This suggested that the OTA-induced inhibition of nucleotide metabolism and enhancement of lipid metabolism were likely dependent on ASK1 activation.

On the other hand, ASK1 partially participated in the regulation of the OTA-induced inhibition of mRNA splicing, the cell cycle and DNA repair. Kuo et al.^70^ reported that Isoobtusilactone A (IOA)-induced cell cycle arrest, associated with the up-regulation of p21 and down-regulation of cyclin B1, cyclin A, cdc2 and cdc25, was regulated by the ROS and ASK1 signaling pathways in human breast cancer cells. Our results further confirmed that ASK1 regulated the OTA-induced cell cycle blockade, coupled with the down-regulation of CDK1 (cdc2). However, the proteomics data showed that ASK1 also has an activating effect on cell cycle progression, along with the down-regulation of STAG2, MKI67 and LIG1 after ASK1 silencing. p38 can function as a dual mediator of ROS signaling and either activate or suppress cell cycle progression depending on the activation stimulus^71^. As ASK1 is the upstream activator of p38, it is reasonable to conclude that ASK1 has a dual role in the cell cycle.

**Figure 10:**
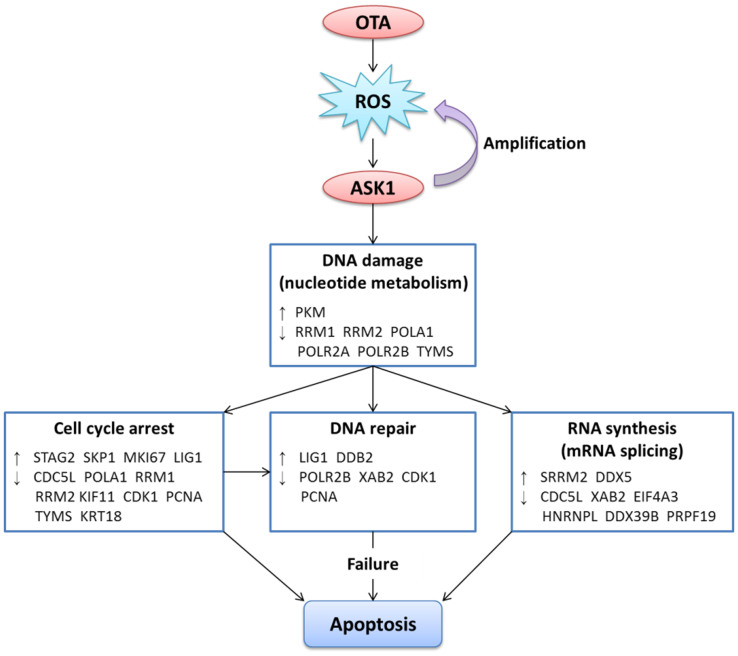
A hypothetical model of ASK1 mediating OTA-induced cytotoxicity. (↑) indicates proteins that were activated or up-regulated and (↓) indicates proteins that were inhibited or down-regulated.

Cell death (typically apoptosis) provoked by genotoxins is largely due to DNA damage, including O^6^-methylguanine, N-methylation lesions, bulky DNA adducts, DNA cross-links and DNA double-strand breaks (DSBs)^72^. Cells respond to DNA lesions by activating cell cycle checkpoint and repair mechanisms; however, failure of the repair system causes tumorigenesis or cell death^73^. At cell cycle checkpoints, including DNA damage, DNA replication and spindle assembly checkpoints, damaged cells are examined, and the cell cycle is arrested until the damage is repaired^74^. If the damage is too serious to repair or the repair system is inhibited, cells undergo apoptosis or other types of cell death^75^,^76^. The inhibition of RNA synthesis is another trigger of cell death upon DNA damage, which leads to a decline in MKP1 (mitogen-activated protein kinase phosphatase 1) and sustained JNK activation^72^. In human fibroblasts, Nucleotide Excision Repair (NER)-defective mutants display sustained and higher levels of JNK activation compared with repair-competent cells following cisplatin treatment^77^. Our results showed that ASK1 regulated the inhibition of DNA repair mechanisms by down-regulating proteins involved in NER (POLR2B, PCNA) and TCR (XAB2), which might further cause prolonged JNK activation and apoptosis initiation. Moreover, protein-protein interactions, including p53 binding to the SH3 domains of some proteins such as Bcl2, play an important role in p53-dependent apoptosis after RNA synthesis is blocked^78^. Altogether, we speculate that the ASK1-mediated blockade of RNA synthesis (predominantly mRNA splicing) can induce apoptosis through the repression of certain genes or through protein-protein interactions. A hypothetical model of the detailed pathway network is shown in Figure 10.

## Conclusion

In summary, our results indicated that OTA activates ASK1 through oxidative stress, whereas ASK1 also has a role in the amplification of ROS generation. Activated ASK1 mediates apoptosis initiation through multiple pathways, including DNA damage (impaired nucleotide metabolism), cell cycle checkpoints, DNA repair mechanisms, and the blockade of RNA synthesis. The cell cycle checkpoints initiate the repair cascade when DNA is damaged. If repair fails, the apoptotic pathway is activated. In the meantime, DNA damage induces the inhibition of RNA synthesis, leading to the repression of certain anti-apoptotic genes. This study shed light on the underlying mechanism by which ASK1 regulates OTA-induced cytotoxicity, providing new ideas for understanding the toxification mechanisms of OTA and the function of ASK1 in mediating cell death.

## Methods

### Cell culture and treatment

HEK293 cells were grown in DMEM (Dulbecco's modified Eagle's medium) supplemented with 10% fetal bovine serum (FBS) (HyClone), 100 U/mL penicillin, 100 μg/mL streptomycin, 250 ng/mL amphotericin B (MacGene, PRC), 2 mM L-Glutamine (Sigma), and 1% Nonessential Amino Acids (MacGene, PRC) at 37°C in a 5% CO_2_ and 95% saturated atmospheric humidity. At approximately 90% confluence, the cells were washed with PBS and then treated with OTA (extracted from corn Aspergillus in our laboratory) or with serum-free medium as a control.

### RNA interference

The ASK1 shRNA and scrambled shRNA were purchased from Santa Cruz Biotechnologies (Santa Cruz Biotechnologies, Dallas, Texas, USA). The ASK1 shRNA Plasmid (h) is a pool of 3 different shRNA plasmids with the following hairpin sequences: GATCCGTACCTCAAGTCTATTGTATTCAAGAGATACAATAGACTTGAGGTACTTTTT, GATCCCAAGGCATTCATACTGAAATTCAAGAGATTTCAGTATGAATGCCTTGTTTTT, and GATCCGGAAGGCTATCATTGACTTTTCAAGAGAAAGTCAATGATAGCCTTCCTTTTT. All sequences are provided in 5′→3′ orientation. shRNA was transfected into cells using VigoFect (Vigorous Biotechnology, PRC) following protocols provided by the manufacturer. Briefly, 2 × 10^5^ cells were seeded into six-well plates and grown to 40–60% confluence. The medium was renewed 1 h before transfection. The mixture of 10 μl of shRNA (1 μg) diluted in 90 μl of normal saline and 1 μl of VigoFect diluted in 99 μl of normal saline was added dropwise to the medium. At 48 h post-transfection, the medium was replaced with fresh selective medium containing 1 μg/mL puromycin (Santa Cruz Biotechnologies) to screen for stably transfected cells. The cultures were replaced with fresh selective medium every two days until no cells were killed.

### Western blotting

Following OTA treatment, HEK293 cells were lysed on ice in RIPA lysis buffer containing 50 mM Tris-HCl (pH 7.4), 1.5 mM NaCl, 1% Triton X-100, 1% sodium deoxycholate, 0.1% SDS and complete protease inhibitor cocktail (sodium orthovanadate, sodium fluoride, EDTA, leupeptin) (Beyotime, PRC) supplemented with 1 mM PMSF. Cells were then homogenized using a 1-mL syringe and centrifuged at 13,000 × g for 10 min at 4°C, as described by Shen^8^. The supernatant was collected and quantified using the BCA Protein Assay Kit (Cwbiotech, PRC). Equal amounts of protein were resolved on a precast 4–10% SDS-PAGE gel and electrotransferred onto a nitrocellulose membrane at 80 V for 2 h. The membrane was then blocked in 1% BSA and Tris-buffered saline containing 0.1% Tween-20 (TBST). After incubating with primary and AP-labeled goat anti-rabbit or anti-mouse secondary antibodies (Beyotime, PRC), specific bands were detected using BCIP/NBT (Merck-Calbiochem). The antibodies used in this study, ASK1 (1:1000), phpspho-ASK1 (1:1000), Actin (1:1000), PKM1/2 (1:1000), cdc2 (1:1000), and KRT8/18 (1:1000), were purchased from Cell Signaling Technology. The DHRS2 antibody (1:200) was purchased from Santa Cruz Biotechnology. The relative intensity of each band was digitized using BandScan V4.3.

### Cell viability assay

The cell viability was assessed by WST-8 staining with the Cell Counting Kit-8 (CCK-8) (Beyotime, PRC) according to the manufacturer's instructions. In brief, 1 × 10^4^ cells were seeded in 96-well plates and treated with 0, 5, 10, 20 or 40 μM of OTA. After incubation for 24 h, 10 μl of WST-8 dye diluted in 100 μl of PBS was added to each well, and the cells were incubated at 37°C for 1 h. The absorbance was read at 450 nm using a Varioskan Flash microplate reader (Thermo Scientific, USA).

### Measurement of intracellular ROS

Intracellular reactive oxygen species (ROS) were detected using the Reactive Oxygen Species Assay Kit (Beyotime, PRC) containing the fluorescent probe 2′,7′-dichloro-fluorescein diacetate (DCFH-DA). In brief, 2 × 10^5^ cells were seeded into six-well plates and incubated for 24 h. After treatment with OTA for 1 h or 24 h, the cells were washed once with PBS and loaded with 10 μM DCFH-DA for 30 min at 37°C in the dark. The formation of the fluorescent-oxidized derivative of DCF-DA was assessed using the FACSCalibur flow cytometer (BD Biosciences, USA). A total of 3 × 10^4^ cells in the gate were collected for flow cytometry analysis.

### Measurement of mitochondrial membrane potential (Δψm)

The mitochondrial membrane potential (Δψm) was measured by 5,50,6,60-tetrachloro-1,10,3,30- tetraethylbenzimidazolycarbocyanine iodide (JC-1, Beyotime, PRC) staining, following the manufacturer's instructions. Briefly, 2 × 10^5^ cells were seeded into a 6-well plate and incubated for 24 h. After OTA treatment, the cells were incubated with JC-1 (Beyotime, PRC) staining solution (5 μg/ml) for 20 min at 37°C. The cells were then washed twice with the JC-1 staining buffer, and the fluorescence intensity of JC-1 monomers (λex = 488 nm, λem = 529 nm) and JC-1 aggregates (λex = 524 nm, λem = 594 nm) was measured using a microplate reader. The Δψm was expressed as the fluorescence ratio of JC-1 aggregates versus JC-1 monomers.

### iTRAQ labeling, mass spectrometry identification and data analysis

#### Protein extraction and iTRAQ labeling

The flow chart of iTRAQ-based proteomics is shown in Figure 6. ASK1 knockdown or scrambled HEK293 cells were treated with or without 20 μM OTA for 24 h. Samples were ground in liquid nitrogen. One milliliter of lysis buffer (7 M urea, 2 M thiourea, 1× Protease Inhibitor Cocktail (Roche Ltd. Basel, Switzerland)) was added to each sample, followed by sonication on ice and centrifugation at 13,000 rpm for 10 min at 4°C. The supernatant was transferred to a fresh tube and stored at −80°C until use. For each sample, proteins were precipitated with ice-cold acetone and then redissolved in dissolution buffer (0.5 M triethylammonium bicarbonate, 0.1% SDS). The proteins were quantified by the BCA protein assay. Then, 100 μg of protein was tryptically digested, and the resultant peptide mixture was labeled using the iTRAQ reagent (Applied Biosystems, California, USA). Briefly, disulfide bonds were reduced in 5 mM Tris-(2-carboxyethy) phosphine (TCEP) at 60°C for 1 h. Cysteine residues were blocked in 10 mM methyl methanethiosulfonate (MMTS) for 30 min at room temperature. The protein solution was further digested with sequence-grade modified trypsin (Promega, Madison, WI). For labeling, each iTRAQ reagent was dissolved in 50 μL of isopropanol and added to the respective peptide mixture. Proteins samples were labeled with iTRAQ isobaric tags 117, 118, 119, and 121, respectively (Figure 6). The labeled samples were combined and dried in vacuo. A SepPac™ C18 cartridge (1 cm^3^/50 mg, Waters Corporation, Milford, MA) was used to remove the salt buffer, and the samples were then dried in a vacuum concentrator for the next step.

#### High pH reverse phase separation

We employed a two-dimensional RP-RP-HPLC separation method with different pHs in two dimensions; this method represents a promising tool for proteomics research and compares favorably with the traditional SCX-RP-HPLC approach in overall performance^79^. In brief, the peptide mixture was redissolved in buffer A (buffer A: 20 mM ammonium formate in water, adjusted with ammonium hydroxide to pH 10.0) and was then fractionated by high pH separation using a Aquity UPLC system (Waters Corporation, Milford, MA) connected to a reverse phase column (XBridge C18 column, 2.1 mm × 150 mm, 3.5 μm, 300 Å, Waters Corporation, Milford, MA). High pH separation was performed using a linear gradient, starting from 5% B and going to 35% B, for 40 min (B: 20 mM ammonium formate in 90% ACN, adjusted with ammonium hydroxide to pH 10.0). The column was re-equilibrated to the initial conditions for 15 min. The column flow rate was maintained at 200 μL/min, and the column temperature was maintained at room temperature. Twelve fractions were collected, and each fraction was dried in a vacuum concentrator before further analysis.

#### Low pH nano-HPLC-MS/MS analysis

The peptides were resuspended with 80 μl solvent C (C: water with 0.1% formic acid; D: ACN with 0.1% formic acid), separated by nanoLC and analyzed by on-line electrospray tandem mass spectrometry. The experiments were performed on a Nano Aquity UPLC system (Waters Corporation, Milford, MA) connected to an LTQ Orbitrap XL mass spectrometer (Thermo Electron Corp., Bremen, Germany) equipped with an online nanoelectrospray ion source (Michrom Bioresources, Auburn, USA). First, 18 μl of each peptide sample was loaded onto the Thermo Scientific Acclaim PepMap C18 column (100 μm × 2 cm, 3-μm particle size), with a flow of 10 μl/min for 5 min, and subsequently separated on the analytical column (Acclaim PepMap C18, 75 μm × 15 cm) with a linear gradient, from 5% D to 45% D, for 165 min. The column was re-equilibrated to the initial conditions for 15 min. The column flow rate was maintained at 300 nL/min, and the column temperature was maintained at 35°C. An electrospray voltage of 1.9 kV versus the inlet of the mass spectrometer was used.

The LTQ Orbitrap XL mass spectrometer was operated in data-dependent mode to automatically switch between MS and MS/MS acquisition^80^. Survey full-scan MS spectra (m/z 400–1600) were acquired in the Orbitrap, with a mass resolution of 30,000 at m/z 400, followed by five sequential HCD-MS/MS scans. The automatic gain control (AGC) was set to 500,000 ions to prevent over-filling of the ion trap. The minimum MS signal for triggering MS/MS was set to 1000. In all cases, one microscan was recorded. MS/MS scans were acquired in the Orbitrap with a mass resolution of 7500. The dissociation mode was higher energy C-trap dissociation (HCD). Dynamic exclusion was used with two repeat counts with a 10-s repeat duration, and the m/z values triggering MS/MS were put on an exclusion list for 120 s. For MS/MS, precursor ions were activated using 40% normalized collision energy and an activation time of 30 ms.

### Database searching and iTRAQ quantification

Protein identification and quantification for the iTRAQ experiment were performed with the ProteinPilot software version 4.0 (Applied Biosystems, California, USA). The database used was the Human UniProtKB/Swiss-Prot database (Release 2012_12_27, with 20233 sequences). The Paragon Algorithm in the ProteinPilot software was used for peptide identification and isoform specific quantification. The data search parameters were set up as follows: trypsin (KR) cleavage with two missed cleavages and fixed modification of cysteines by MMTS were considered, and iTRAQ modification of peptide N termini, methionine oxidation and iTRAQ modification of lysine residues were set as variable modifications. To minimize false positive results, a strict cutoff for protein identification was applied with the unused ProtScore ≥ 1.3, which corresponds to a confidence limit of 95%, and at least two peptides with 95% confidence were considered for protein quantification. The resulting data set was automatically bias-corrected to remove any variations imparted due to unequal mixing during the combination of different labeled samples. For iTRAQ quantitation, the peptide for quantification was automatically selected by the Pro Group algorithm (at least two peptides with 99% confidence) to calculate the reporter peak area, error factor (EF), and p-value. For the selection of differentially expressed proteins, the proteins had to contain at least two unique high-scoring peptides.

### Analysis of proteomics data

The differentially expressed proteins were analyzed according to the functional annotations from the UniProt knowledge base (Swiss-Prot/TrEMBL, http://www.uniprot.org/) and GO database (http://www.geneontology.org/). The metabolic pathways associated with the differentially expressed proteins were classified using KEGG_PATHWAY, PANTHER_PATHWAY and REACTOME_PATHWAY in the DAVID database (http://david.abcc.ncifcrf.gov/).

### Statistical analysis

Microsoft Excel 2010 and SPSS 20 (SPSS, Inc) were used for the statistical analysis. Data were subjected to an analysis of variance (ANOVA), and a comparison of means was carried out by Duncan's multiple-range test. *p* < 0.05 is considered to be statistically significant.

## Figures and Tables

**Table 1:** Proteins differentially expressed in response to OTA treatment versus control in Scrambled shRNA cell group (118:117). Shading area indicates proteins that also appeared in ASK1 knockdown cell group. Proteins are ordered alphabetically by their gene names

UniProtKB Accession	Gene name	Protein name	Unused protein score	% of Coverage	No. of unique Peptides	Ratio(118:117) Mean ± SD
Q13085	ACACA	Acetyl-CoA carboxylase 1	3.47	1.194	3	1.237 ± 0.069
P02768	ALB	Serum albumin	15.55	12.97	9	2.521 ± 0.311
Q99459	CDC5L	Cell division cycle 5-like protein	17.74	12.84	9	0.764 ± 0.052
P06493	CDK1	Cyclin-dependent kinase 1	16.2	29.97	8	0.646 ± 0.025
Q13838	DDX39B	Spliceosome RNA helicase DDX39B	34.27	37.85	20	0.777 ± 0.011
P17844	DDX5	Probable ATP-dependent RNA helicase DDX5	15.28	29.97	21	1.228 ± 0.056
Q13268	DHRS2	Dehydrogenase/reductase SDR family member 2	12.05	29.07	6	0.783 ± 0.050
P38919	EIF4A3	Eukaryotic initiation factor 4A-III	23.75	46.23	17	0.811 ± 0.001
Q96AY3	FKBP10	Peptidyl-prolyl cis-trans isomerase FKBP10	20.49	18.56	10	1.369 ± 0.073
Q8N1G2	FTSJD2	Cap-specific mRNA (nucleoside-2'-O-)-methyltransferase 1	8.78	4.311	4	0.828 ± 0.049
P14866	HNRNPL	Heterogeneous nuclear ribonucleoprotein L	23.6	25.98	15	0.767 ± 0.010
Q13123	IK	Protein Red	4.56	3.052	2	0.706 ± 0.032
P52732	KIF11	Kinesin-like protein KIF11	13.19	7.955	6	0.830 ± 0.006
Q9BTE3	MCMBP	Mini-chromosome maintenance complex-binding protein	10.53	7.477	5	0.801 ± 0.024
P07237	P4HB	Protein disulfide-isomerase	41.66	40.75	21	1.240 ± 0.043
P12004	PCNA	Proliferating cell nuclear antigen	23.6	50.57	12	0.652 ± 0.025
P14618	PKM	Pyruvate kinase isozymes M1/M2	74.85	67.98	44	1.249 ± 0.034
P13797	PLS3	Plastin-3	59.77	51.27	37	1.205 ± 0.033
P09884	POLA1	DNA polymerase alpha catalytic subunit	15.9	6.293	7	0.829 ± 0.021
P24928	POLR2A	DNA-directed RNA polymerase II subunit RPB1	10.68	3.401	5	0.697 ± 0.004
P30876	POLR2B	DNA-directed RNA polymerase II subunit RPB2	20.98	11.07	12	0.828 ± 0.007
Q9UMS4	PRPF19	Pre-mRNA-processing factor 19	17.83	22.22	10	0.759 ± 0.067
Q15293	RCN1	Reticulocalbin-1	13.59	25.68	8	1.321 ± 0.010
P05388	RPLP0	60S acidic ribosomal protein P0	29.66	57.41	16	1.209 ± 0.012
P23921	RRM1	Ribonucleoside-diphosphate reductase large subunit	23.29	14.39	11	0.812 ± 0.049
P31350	RRM2	Ribonucleoside-diphosphate reductase subunit M2	14.35	22.11	7	0.778 ± 0.031
P22307	SCP2	Non-specific lipid-transfer protein	13.32	12.43	8	1.214 ± 0.046
Q8NC51	SERBP1	Plasminogen activator inhibitor 1 RNA-binding protein	12.32	15.93	7	1.283 ± 0.060
P35237	SERPINB6	Serpin B6	13.45	27.93	7	1.244 ± 0.030
Q2TAY7	SMU1	WD40 repeat-containing protein SMU1	9.75	10.53	7	0.696 ± 0.035
Q9UQ35	SRRM2	Serine/arginine repetitive matrix protein 2	17.08	5.16	9	1.207 ± 0.052
P04818	TYMS	Thymidylate synthase	8.04	15.34	4	0.290 ± 0.046
Q9HCS7	XAB2	Pre-mRNA-splicing factor SYF1	6.47	3.743	3	0.512 ± 0.073

**Table 2:** Proteins differentially expressed in response to OTA treatment versus control in ASK1 knockdown cell group (121:119). Shading area indicates proteins that also appeared in scrambled cell group. Proteins are ordered alphabetically by their gene names

Accession	Gene name	Protein name	Unused protein score	% of Coverage	No. of unique Peptides	Ratio (121:119) Mean ± SD
P06493	CDK1	Cyclin-dependent kinase 1	16.2	29.97	8	0.732 ± 0.011
P00387	CYB5R3	NADH-cytochrome b5 reductase 3	19.51	42.52	10	1.202 ± 0.016
Q92466	DDB2	DNA damage-binding protein 2	8.67	12.18	4	0.809 ± 0.058
Q13838	DDX39B	Spliceosome RNA helicase DDX39B	34.27	37.85	20	0.802 ± 0.025
P17844	DDX5	Probable ATP-dependent RNA helicase DDX5	15.28	29.97	21	1.204 ± 0.038
Q13268	DHRS2	Dehydrogenase/reductase SDR family member 2	12.05	29.07	6	0.753 ± 0.037
Q96AY3	FKBP10	Peptidyl-prolyl cis-trans isomerase FKBP10	20.49	18.56	10	1.263 ± 0.019
O75367	H2AFY	Core histone macro-H2A.1	24.18	44.62	18	1.225 ± 0.034
Q13123	IK	Protein Red	4.56	3.052	2	0.668 ± 0.033
P05783	KRT18	Keratin, type I cytoskeletal 18	40.25	55.81	21	1.299 ± 0.076
P05787	KRT8	Keratin, type II cytoskeletal 8	38.34	52.59	28	1.212 ± 0.003
P18858	LIG1	DNA ligase 1	11.74	5.767	6	0.824 ± 0.029
P49257	LMAN1	Protein ERGIC-53	7.28	14.12	4	1.231 ± 0.014
Q9BTE3	MCMBP	Mini-chromosome maintenance complex-binding protein	10.53	7.477	5	0.822 ± 0.035
P46013	MKI67	Antigen KI-67	21.5	6.665	11	0.821 ± 0.003
O94776	MTA2	Metastasis-associated protein MTA2	10.88	9.431	6	0.782 ± 0.017
P12004	PCNA	Proliferating cell nuclear antigen	23.6	50.57	12	0.681 ± 0.026
P24928	POLR2A	DNA-directed RNA polymerase II subunit RPB1	10.68	3.401	5	0.783 ± 0.041
Q13162	PRDX4	Peroxiredoxin-4	18.46	51.66	18	1.262 ± 0.081
Q9UMS4	PRPF19	Pre-mRNA-processing factor 19	17.83	22.22	10	0.822 ± 0.014
P63208	SKP1	S-phase kinase-associated protein 1	4.89	16.56	3	0.756 ± 0.038
Q2TAY7	SMU1	WD40 repeat-containing protein SMU1	9.75	10.53	7	0.697 ± 0.032
Q8N3U4	STAG2	Cohesin subunit SA-2	12.78	5.605	7	0.822 ± 0.060
P04818	TYMS	Thymidylate synthase	8.04	15.34	4	0.376 ± 0.038
